# Distribution and protective function of pituitary adenylate cyclase-activating polypeptide in the retina

**DOI:** 10.3389/fendo.2012.00145

**Published:** 2012-11-23

**Authors:** Tomoya Nakamachi, Attila Matkovits, Tamotsu Seki, Seiji Shioda

**Affiliations:** ^1^Department of Anatomy, Showa University School of MedicineTokyo, Japan; ^2^Center for Biotechnology, Showa UniversityTokyo, Japan; ^3^Department of Anatomy, University of PécsPécs, Hungary; ^4^Department of Ophthalmology, Showa University School of MedicineTokyo, Japan

**Keywords:** PACAP, PACAP receptor, retina, distribution, protection, rodent, review, knockout mouse

## Abstract

Pituitary adenylate cyclase-activating polypeptide (PACAP), which is found in 27- or 38-amino acid forms, belongs to the VIP/glucagon/secretin family. PACAP and its three receptor subtypes are expressed in neural tissues, with PACAP known to exert a protective effect against several types of neural damage. The retina is considered to be part of the central nervous system, and retinopathy is a common cause of profound and intractable loss of vision. This review will examine the expression and morphological distribution of PACAP and its receptors in the retina, and will summarize the current state of knowledge regarding the protective effect of PACAP against different kinds of retinal damage, such as that identified in association with diabetes, ultraviolet light, hypoxia, optic nerve transection, and toxins. This article will also address PACAP-mediated protective pathways involving retinal glial cells.

## INTRODUCTION

Pituitary adenylate cyclase-activating polypeptide (PACAP) is a neuropeptide which was originally isolated from ovine hypothalamus on the basis of its capacity to stimulate adenylate cyclase activity in pituitary cells (Miyata et al., 1989). PACAP exists in two forms containing 27 and 38 amino acid residues, respectively, with PACAP27 sharing 68% sequence homology with that of vasoactive intestinal polypeptide (VIP). PACAP belongs to the VIP/secretin/glucagon family of peptides (Arimura and Shioda, 1995). PACAP38 is the predominantly expressed form in mammals (Arimura et al., 1991). The amino acid sequence of PACAP has been well conserved in vertebrates, implying that PACAP may act as an important neuropeptide (Sherwood et al., 2000). The receptors for PACAP, of which there are three main types, belong to the G protein-coupled receptor family with seven transmembrane domains. The PAC1 receptor (PAC1R) binds PACAP with high affinity and VIP with a much lower affinity, while the VPAC1 and VPAC2 receptors (VPAC1R, VPAC2R) bind VIP and PACAP with similar affinities (Harmar et al., 2012). PACAP is a pleiotropic biological peptide which regulates vasodilation, activates intestinal motility, increases insulin and histamine secretion, and modulates immune responses in peripheral tissues (Vaudry et al., 2009). In the central nervous system (CNS), PACAP acts as a neurotransmitter, neuromodulator, and/or neurotrophic factor (Arimura, 1998; Nakamachi et al., 2011). One of the most important functions of PACAP is that of neuroprotection. In this way, PACAP suppresses neuronal damage against acute brain injuries such as brain ischemia, traumatic brain injury, and spinal cord injury (Farkas et al., 2004; Ohtaki et al., 2008; Tsuchikawa et al., 2012). Moreover, PACAP protects the brain against neurodegenerative disease (Reglodi et al., 2011), reducing the level of neuronal damage. The retina is considered as a specialized neuronal tissue containing different types of neurons and glial cells. Therefore, to reveal any functions of PACAP in this tissue, it is necessary to understand which cell types in the retina express PACAP and PACAP receptors. The purpose of this review is to provide an overview of the distribution of PACAP and its receptors in the retina, and to summarize current knowledge of the protective functions of PACAP in animal models of retinopathy.

## EXPRESSION OF PACAP AND ITS RECEPTORS IN RETINAL TISSUE

### PACAP DISTRIBUTION

From a morphological perspective, light microscopy immunohistochemistry studies have shown PACAP-like immunoreactivity (-LI) exists in a population of sensory neurons in the rat uvea (Moller et al., 1993; Mulder et al., 1994) and in rabbit trigeminal ganglion cells (Onali and Olianas, 1994). Nerve fibers with PACAP-LI have also been found in the uvea of the rat eye (Moller et al., 1993; Mulder et al., 1994). PACAP-LI in the eye was studied by radio-immunoassay and the highest concentrations were found in the iris sphincter and ciliary body (Onali and Olianas, 1994; Wang et al., 1995). Furthermore, immunohistochemical studies revealed that PACAP-positive nerve fibers were present in the nerve fiber layer (NFL), the ganglion cell layer (GCL), and the inner plexiform layer (IPL). PACAP-positive neuronal cell bodies were also found in amacrine and horizontal cells in the inner nuclear layer (INL). No PACAP-LI was found in photoreceptors in the outer nuclear layer (ONL) or retinal pigmented epithelium (Seki et al., 1997, 1998; Izumi et al., 2000).

At the ultrastructural level, PACAP-LI was found in some amacrine and horizontal cells in the INL. PACAP-LI was detected in the plasma membrane and rough endoplasmic reticulum, and diffusible immunoreactive products were detected in the cytoplasmic matrix of amacrine cells and horizontal cells. Intense PACAP-LI was detected in cell processes of the IPL, GCL, and NFL. In the IPL, PACAP-positive amacrine cell processes make synaptic contacts with retinal ganglion cell (RGC) terminals, as well as amacrine and bipolar cell processes. PACAP-positive amacrine cell processes have also been identified to make synaptic contacts with each other. In the IPL, PACAP-positive presynaptic axon terminals were found to contain several dense granular vesicles (80–100 nm in diameter) and many small clear synaptic vesicles (30–50 nm in diameter). However, precise ultrastructural localization of PACAP-LI in the axon terminals is very difficult to determine with the pre-embedding immunostaining method. Electron microscopy observations of the IPL revealed non-specific immunoreactive products associated with post-synaptic membranes, the outer mitochondrial membrane, and the cytoplasmic matrix of axon terminals (Seki et al., 2000b). PACAP-LI is also expressed in the cell bodies of some amacrine and horizontal cells in the INL and their processes in the IPL, GCL, and NFL in the rat retina, as shown by both light and electron microscopic immunocytochemistry. PACAP-positive axon terminals make synaptic contact with RGC, bipolar cells, amacrine cells, and horizontal cells in the GCL, NFL, and IPL. On the other hand, VIP-positive cells have also been found in the GCL and INL, and their fibers have been found in the IPL (Loren et al., 1980). Both PACAP- and VIP-positive cells and fibers are found in the rat retina but their distributions are quite different from each other. These studies strongly suggest that PACAP and VIP function as neurotransmitters and/or neuromodulators, but the functions of PACAP may be different from those of VIP.

### PACAP RECEPTOR DISTRIBUTION

**FIGURE 1:**
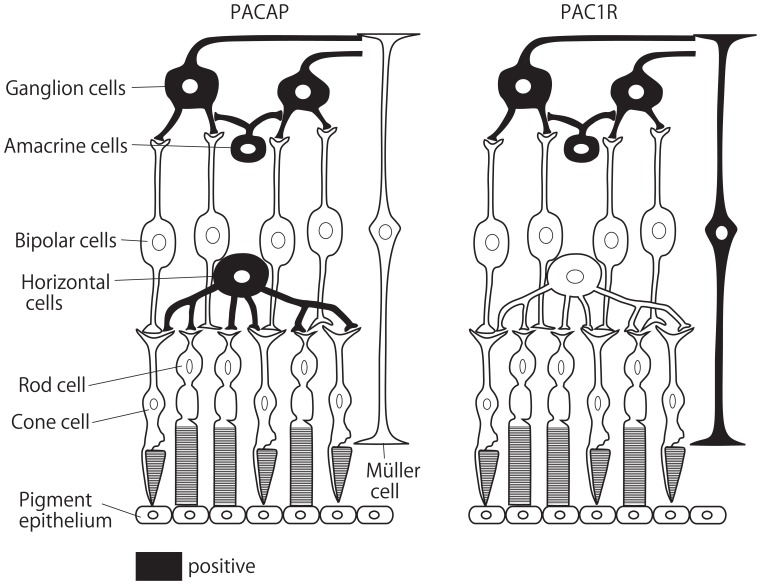
**Schematic diagram of PACAP and PAC1R distributions in the rodent retina**. Black color indicates PACAP or PAC1R-expressing cells according to previous reports.

Receptor binding sites for PACAP and VIP, positively coupled to adenylate cyclase, have been previously described in the retina of different mammalian species (D’Agata and Cavallaro, 1998). As to the localization of PAC1R in the rat retina (Nilsson et al., 1994), we have described the distribution and localization of PAC1R and its mRNA in the rat retina by immunohistochemistry and *in situ* hybridization histochemistry (Seki et al., 1997, 2000a). PAC1R-LI was found in the cell bodies and processes of RGC and amacrine cells. No PAC1R-LI was observed in photoreceptors. At the ultrastructural level, PAC1R-LI was detected in the plasma membrane, rough endoplasmic reticulum, and the cytoplasmic matrix of RGCs and amacrine cells in the INL. There are certain areas in which the localization of PACAP does not match that of PAC1R. For example, in the rat brain, PAC1R has been found at very high levels in the olfactory bulb, hippocampus, and cerebellar cortex, where few PACAP-containing neurons are identified (Seki et al., 1997). Reports also suggest that PACAP is a transmitter and/or modulator which regulates RGCs and amacrine cells in the rat retina. Müller cells were difficult to identify in histological observations, but PAC1R-LI was observed in rat primary cultures of Müller cells (Seki et al., 2006a). PACAP and PAC1R distributions in the rodent retina are summarized in Figure 1.

## PACAP AND NEUROPROTECTION

Protective effect of PACAP on retina and retinal cells against various types of retinopathy animal models and toxic reagent showing below was summarized in Table 1.

**Table 1:** Summary of current knowledge on the effects of PACAP in retinal cells.

Toxin/model	Treatment of PACAP			Reference
	Tissue/cells	Dose	Effect	
***In vitro***
Glutamate (1 mM)	Retinal tissue culture from rat pups	PACAP27 or 38 (10 nM to 1 µM)	Protect neuronal cells (maximum effect at 1 µM)	Shoge et al. (1999)
Thapsigargin (10 nM)	Retinal tissue culture from rat pups	PACAP38 (1 nM)	Protect photoreceptor cell	Silveira et al. (2002)
Anisomycin (1 µg/ml)	Retinal tissue culture from rat pups	PACAP38 (100 fM to 10 nM)	Protect neuroblastic layer (maximum effect at 1 nM)	Silveira et al. (2002)
Anoxic condition	Turtle eyecup preparation	PACAP38 (0.165 µM)	Protect horizontal cells	Rabl et al. (2002)
	Primary culture of rat Müller cell	PACAP38 (1 pM to 1 µM)	Increase IL-6 levels	Nakatani et al. (2006)
H_2_O_2_ (0.2–0.3 mM)	ARPE19 human retinal pigment epithelium	PACAP38 (10 pM to 1 µM)	Increase the number of surviving cells (maximum effect at 100 nM)	Mester et al. (2011)
***In vivo***
MSG (2 mg/g, SC, three times)	Rat retina (PD21)	PACAP27 or 38 (100 pmol, IVI)	Protect INL, IPL, and GCL	Tamas et al. (2004) Babai et al. (2005), 2006
Kainic acid (5 nmol, IVI)	Rat retina (adult)	PACAP38 (100 pmol, IVI)	Protect GCL	Seki et al. (2006a)
BCCAO (permanent occlusion)	Rat retina (adult)	PACAP38 (100 pmol, IVI)	Protect GCL, OPL, and INL	Atlasz et al. (2007)
Optic nerve transection	Rat retina (adult)	PACAP38 (1 pM to 1 nM, 3 µl)	Protect GCL (maximum effect at 10 and 100 pM)	Seki et al. (2008)
UV-A irradiation	Rat retina (adult)	PACAP38 (100 pmol, IVI)	Protect ONL and GCL	Atlasz et al. (2011)
High intraocular pressure	Rat retina (adult)	PACAP38 (1 fM to 1 nM, 3 µl IVI)	Protect GCL (bimodal effect at 10 fM and 10 pM)	Seki et al. (2011)
NMDA (40 nmol, IVI)	PACAP^+/–^ and PACAP^+/+^ mice retina (adult)	PACAP38 (10 fM to 10 nM, 2 µl IVI)	Protect GCL (maximum effect at 100 pmol)	Endo et al. (2011)
MSG (2 mg/g, SC, three times)	Rat retina (PD21)	PACAP38 (100 pmol, IVI)	Retinal damage in PACAP^–/–^ mice was more severe in GCL	Kiss et al. (2011) Varga et al. (2011)
BCCAO (10 min occlusion)	PACAP^–/–^ and PACAP^+/+^ mice retina (adult)	PACAP38 (100 pmol, IVI)	Protect all layers of the retina, and improve ERG response	Szabadfi et al. (2012a)
Streptozotocin (70 mg/kg i.p.)	Rat retina (adult)	PACAP38 (100 pmol, IVI)	Retinal damage in PACAP^–/–^ mice was more severe in all layers	Szabadfi et al. (2012b)

*BCCAO, bilateral common carotid artery occlusion; ERG, electroretinography; GCL, ganglion cell layer; INL, inner nuclear layer; IPL, inner plexiform layer; IVI, intravitreal injection; MSG, monosodium glutamate; NMDA, *N*-methyl-refscd-aspartate; ONL, outer nuclear layer; OPL, outer plexiform layer; PD, postnatal day; SC, subcutaneous injection; UV, ultraviolet.

### PACAP AND DIABETES (DIABETIC RETINOPATHY)

It is well known that diabetes causes numerous health complications in the human body, with one of the most serious consequences of this disease being diabetic retinopathy. The reason for the retinal degeneration is that the retina is unable to adapt to the metabolic changes caused by hyperglycemia. This situation leads to chronic inflammation and microvascular angiopathy (Liu et al., 2008). One of the most important consequences of diabetes is the changes to enzyme activities and altered expression patterns of growth and transcription factors (Seki et al., 2004). Another reason for the degeneration of the retina is related to oxidative stress (Kowluru and Abbas, 2003). Increased production of reactive oxygen species (ROS) may play an important role in the development of diabetic complications as it has been shown that ROS levels are elevated in the diabetic rat retina and in retinal cells incubated in high glucose media (Kowluru and Abbas, 2003; Cui et al., 2006) due to the hyperglycemia-induced impairment of antioxidant defense systems (Kowluru et al., 1996). The progression of diabetic retinopathy is slow, and leads to a decrease in a number of cell types in the retina such as amacrine cells, RGCs, and both types of photoreceptors (rod and cone cells; Holopigian et al., 1997; Gastinger et al., 2006). Müller cells and retinal astrocytes are also affected, becoming reactive within 3 months of the onset of diabetes (Lieth et al., 1998). Increased glial fibrillary acidic protein (GFAP) expression has been reported, particularly in the early phase of the disease (Lieth et al., 1998), which is closely associated with the activation of Müller cells.

Some studies have shown that PACAP treatment could protect the retina against the harmful effects of diabetes. In streptozotocin-treated rats, PACAP (100 pmol in 5 µl saline) administered into the vitreous body three times over the course of a week was able to attenuate the decrease of the cell number in the GCL (Szabadfi et al., 2012b). The PACAP treatment was also effective with respect to Müller cells in that the streptozotocin injection increased GFAP immunoreactivity in the retina due to Müller cell activation, but the PACAP treatment was able to significantly decrease the number of GFAP-positive Müller cells compared with untreated animals (Szabadfi et al., 2012b).

The PACAP treatment also increased the expression pattern of PAC1R in the diabetic retina, particularly in tyrosine-hydroxylase (TH)-positive cells (Szabadfi et al., 2012b). The most important TH-positive cells in the retina are the amacrine cells. The morphological or functional degeneration of dopaminergic amacrine cells was observed during the early stage of STZ-induced diabetes (Seki et al., 2004), and it was recently confirmed immunohistochemically that intravitreally injected PACAP is able to protect the amacrine cells from such degeneration (Atlasz et al., 2010b). Diabetes also causes changes in the expression of several apoptotic factors. Three weeks after streptozotocin injection, B-cell lymphoma 2 (Bcl-2) expression was decreased and p53 expression was increased, both of which indicate an elevated level of apoptosis of the retinal cells. The intravitreal injection of PACAP (100 µmol in 5 µl of saline) was able to block these changes and restore the Bcl-2 and p53 levels to near control (Giunta et al., 2012). These results suggest that PACAP is able to up-regulate anti-apoptotic pathways and down-regulate pro-apoptotic pathways.

### PACAP AND ULTRAVIOLET-A LIGHT-INDUCED RETINOPATHY

Only one paper has been published concerning the capacity of PACAP to protect against ultraviolet (UV) light-induced retinopathy. Exposure of animals for 45 min to diffuse UV-A irradiation caused cell death especially in the ONL and INL of the retina, and the thickness of the retina decreased in line with the duration of the UV-A treatment. One day after the UV-A treatment the GCL was not affected, but following a second day after illumination a significant decrease of the number of RGCs was observed. The intravitreal administration of PACAP (100 pmol in 5 µl saline) immediately after the irradiation was able to protect the ONL and INL of the retina which are seriously affected by the UV-A light, and the number of the cells in INL, ONL, and GCL was significantly increased as a consequence of the PACAP treatment (Atlasz et al., 2011). This effect of PACAP may have been via a reduction in the level and toxicity of free radicals generated as a consequence of the exposure to the UV-A. PACAP may thus reduce UV-A radiation-induced retinal damage and edema in a manner similar to that in which it reduces ischemia-induced cerebral damage and edema (Ohtaki et al., 2008; Nakamachi et al., 2010).

### PACAP AND HYPOXIA

The retina has an elevated oxygen uptake compared to other tissues, making it one of the most sensitive tissues in the human body to hypoxia. Several diseases can cause retinal ischemia, the most common of which are cardiovascular disorders such as carotid artery stenosis, retinal artery occlusion, diabetic retinopathy, or high intraocular pressure, which can compress the blood vessels of the retina and cause hypoperfusion (Seki et al., 2011). An optimal way to model retinal ischemia in rats is the bilateral common carotid artery occlusion (BCCAO) technique; however the outcome of the operation may depend on the rat strain and technique used (Block et al., 1992; Szabadfi et al., 2010). The permanent ligation of both common carotid arteries leads to a reduction in the cerebral blood flow in rats (Farkas et al., 2005; Lavinsky et al., 2006), which can cause severe retinal damage. All layers of the retina can be affected, with the reduction of the blood flow usually leading to a decrease of the thickness of the retina, especially with respect to the inner and outer plexiform layers (Atlasz et al., 2007; Atlasz et al., 2010b). The photoreceptors can also be damaged, with their outer segments becoming shorter, and alterations to their structure appearing after the BCCAO (Atlasz et al., 2010b).

In recent years, several studies have been published concerning retinoprotective strategies and agents developed to combat retinal ischemia. Because decreased perfusion leads to a range of changes in the retina, such as an altered metabolism of glutamate, increased levels of ROS, mitochondrial failure, and activation of inflammatory mediators, several possibilities to reduce the harmful effects of ischemia have been proposed (Szabadfi et al., 2010). For example, numerous retinoprotective agents or methods have been tested in the last few years against the harmful effects of retinal ischemia; these include VIP, poly ADP-ribose polymerase (PARP) inhibitors, brain-derived neurotrophic factor (BDNF), antioxidants, flavonoids, pre- or post-conditioning, etc. (Atlasz et al., 2010a; Szabadfi et al., 2010). Both *in vitro* and *in vivo* studies have shown PACAP to be one of the best candidates to protect retinal cells and to reduce the effects of ischemia. In an early report, turtle retina fragments were maintained in non-oxygenated Ringer solution for 46 h, with added PACAP38 (0.165 µM) able to protect the horizontal cells against ischemia; after 42 and 46 h, the light response of the cells was significantly higher than responses obtained from control group fragments (Rabl et al., 2002).

Several papers were published thereafter concerning the retinoprotective action of PACAP against the effects of hypoxia. PACAP possibly acts via PAC1R, which is detectable in all layers of the retina and is strongly expressed in the GCL, INL, NFL, and more weakly in the IPL, OPL, and ONL (Seki et al., 1997, 2000a). PACAP (10 pmol in 5 µl saline) intravitreally administered immediately after the BCCAO operation significantly reduced the harmful effects of ischemia compared to sham-operated animals. This protective effect was significantly attenuated by the PACAP38 antagonist, PACAP6-38 (Atlasz et al., 2007). Several cell types in the retina can be damaged by ischemia. A decrease of vesicular glutamate transporter 1 (VGLUT1) transporters causes damage to photoreceptors, bipolar cells, and calcium binding proteins, giving rise to the degeneration of different types of neurons. Moreover, increased GFAP expression is a sign of Müller cell and astrocyte activation. These effects were attenuated by PACAP treatment after the BCCAO (Atlasz et al., 2010b), suggesting that PACAP has a general cytoprotective effect in the retina against hypoxic conditions.

This effect of PACAP38 was confirmed in another study on wild-type and PACAP-null CD1 mice exposed to transient (10 min) BCCAO. Directly after the operation PACAP38 (100 pmol in 3 µl saline) was administered into the vitreous body. The results of the operation and treatment were tested 2 weeks later. The 10-min BCCAO resulted in a thinner retina, with significantly greater damage evident in the PACAP-null animals. In this group all the retinal layers were affected, while in the wild-type animal abnormalities were only evident in the INL. Intravitreal PACAP38 treatment significantly attenuated the deleterious effects of BCCAO in both groups. These results suggest that the retina in PACAP-null animals is more sensitive to ischemia compared to that in wild-type mice, and that PACAP treatment is effective against retinal ischemia in both wild-type and PACAP-null animals (Szabadfi et al., 2012a).

Another technique to transiently decrease retinal blood flow is to artificially elicit intraocular hypertension. This is a glaucoma model, where a thin needle is inserted into the anterior chamber of the eye of adult rats and 0.9% saline is injected to temporarily increase the intraocular pressure up to 100 mmHg for 60 min. The result of this procedure is similar to that achieved with BCCAO, in that under these experimental conditions the intraocular pressure is greater than the blood pressure in the vessels of the retina, thereby causing a hypoxic state in the retina. This treatment leads to a decrease in the number of RGCs and in the thickness of the retina, especially the IPL (Seki et al., 2011). PACAP38 treatment was also effective in combating this situation given that, compared with untreated eyeballs; the number of the RGCs was higher in animals treated with PACAP38. The effect of the PACAP was significantly stronger compared to the vehicle-treated animals when 3 µl of PACAP38 solution at concentrations of 10 fM, 10 or 100 pM was administered. This result suggests that the PACAP has a bimodal effect with peaks at 10 fM and 10–100 pM, even though the mechanism accounting for these effects was not the same. In the case of the 10 fM PACAP administration, the mitogen-activated protein kinase (MAPK) inhibitor PD-98059 significantly reduced the protective effect of PACAP, but there was no significant difference observed at the other PACAP concentrations tested. Meanwhile, the cAMP antagonist Rp-cAMP significantly decreased the effect of PACAP at all the concentrations tested (Seki et al., 2011). These results suggest that pathways involving MAPK and cAMP play key roles in the protective effect of PACAP against hypoxia. A recent study revealed additional information concerning this phenomenon. Adult rat retinas were examined 5, 30, and 60 min after BCCAO accompanied by injections of 100 nmol/3 µl of PACAP. The results showed that in the absence of any harmful stimulus, the PACAP did not cause any changes compared with the control group. Ischemia itself caused several changes, such as increases in the phosphorylation of Akt, ERK 1/2, JNK, or p38MAPK, particularly at the 30 and 60 min time points following the operation. PACAP treatment caused a significant increase in the phosphorylation of Akt and ERK 1/2 at all time points, and decreased the activity of JNK and p38MAPK (Szabo et al., 2012). Several results were published previously concerning the effect of PACAP on these signal transduction pathways in the retina and other tissues (Dohi et al., 2002; Ohtaki et al., 2006; Racz et al., 2006; Shioda et al., 2006). PACAP has important, but variable, effects on the expression of interleukins and cytokines. The expression of several interleukins such as inter interleukin (IL)-1, intercellular adhesion molecule (ICAM), L-selectin, regulated and normal T cell expressed and secreted (RANTES) etc, was decreased by PACAP, while levels of others such as IL-2, IL-6, IL-10, and tumor necrosis factor (TNF)-α were not changed. In contrast, the levels of vascular endothelial growth factor (VEGF) and thymus chemokine were increased (Szabo et al., 2012).

Not only ischemia, but also hyperoxia cause oxidative stress which could be harmful for cells in the retina. Oxidative stress is one of the most important apoptosis-inducing factors in the human body, especially in the CNS and sensory organs (Vaudry et al., 2002; Racz et al., 2010). Retinal pigment epithelial cells are sensitive to oxidative stress, with hydrogen peroxide- or aldehyde-induced oxidative stress leading to apoptosis of these cells *in vitro* (Kalariya et al., 2008; Kook et al., 2008). Given that retinal pigment epithelial cells express PAC1R and VPAC receptors (Zhang et al., 2005), PACAP could have a cytoprotective effect on these cells. When retinal pigment epithelial cells were treated with 0.25 mM H_2_O_2_ and 10 nM PACAP38 for 3 h, the survival of these cells was significantly ameliorated compared with control cells not treated with PACAP. An MAPK inhibitor in this case had no influence on the effect of PACAP38, but inhibition of the phosphoinositide 3-kinase (PI3K)/Akt pathway and PACAP6-38 treatment were able to antagonize this cytoprotective effect (Mester et al., 2011). The p38MAPK, c-Jun N-terminal kinase (JNK), extracellular signal-regulated kinase (ERK) 1/2, and Akt pathways were also activated in response to H_2_O_2_ treatment, while PACAP38 decreased the level of p38MAPK and pJNK, and increased the activity of the ERK 1/2 and Akt pathways. Other cytokine and signal transduction pathways are modified by H_2_O_2_ and PACAP38 treatment. Oxidative stress induced the expression of several apoptosis-inducing factors such as Bad, Bax, Trail, Fas-associated protein with death domain (FADD), Fas, second mitochondrial-derived activator of caspase (SMAC), and several heat-shock proteins (HSP32, HMOX2, and HSP27), as well as p53. Co-treatment with both 100 and 10 nM PACAP38 decreased the activation all of these factors (Fabian et al., 2012).

### PACAP AND OPTIC NERVE TRANSECTION

Optic nerve injury caused by trauma, glaucoma, or neurodegenerative disease can cause apoptotic RGCs death (Quigley et al., 1995), for which, based on the above findings, PACAP could be a good candidate to protect injured RGCs against apoptosis. In one recent study, different concentrations of PACAP38 were administered into the vitreous body of adult rats, and immediately after the injection, the optic nerve was transected (Seki et al., 2008). Fourteen days later the number of the RGCs was found to be significantly decreased in the control (untreated) and PACAP-treated groups due to apoptosis. The PACAP38 treated groups, however, showed increased RGC survival compared with control, particularly with regard to the groups receiving injections of 3 µl of saline containing 10 and 100 pM PACAP.

### PACAP AND TOXINS

Many toxic agents have been identified which can cause severe retinal injury. Some of them, like glutamate, are found in the retinal cells and are necessary in small concentrations for the normal functioning of the retina. Others, like anisomycin and thapsigargin, are not endogenous to the retina and are commonly used in *in vivo* studies to induce retinal injury by direct administration into the vitreous body.

#### Anisomycin

The capacity of PACAP to prevent the deleterious effects of this drug was examined on *in vitro* preparations of the retinal neuroblastic layer from newborn rats. This drug inhibits protein synthesis and causes cell death in the neuroblastic layer. It was previously found that increased cAMP levels protect retinal cells against retinal damage induced by protein synthesis inhibition (Rehen et al., 1996). In this way, 1 nM PACAP38 or PACAP27 administered in parallel with anisomycin had a protective effect on the neuroblastic layer (Silveira et al., 2002). PACAP38 exerts its action via the PAC1 receptor, which is expressed in all layers of the neonatal retina, and activates the cAMP/protein kinase A (PKA) pathway, which is essential for this effect. The activation of other PAC1R-activated signal transduction pathways, such as the phospholipase C (PLC) or PI3K pathways, is not necessary for the protective effect of PACAP against anisomycin (Silveira et al., 2002). On the other hand, PACAP6-38 and Maxadilan (a specific PAC1R antagonist) inhibit this neuroprotective effect of PACAP38 (Silveira et al., 2002).

#### Thapsigargin

Thapsigargin is a non-competitive inhibitor of the endoplasmic reticulum Ca^2^^+^-ATPase. This drug inhibits autophagia, which leads to cell death. In the case of retinal explants of newborn rats, thapsigargin causes apoptosis of the photoreceptors in the ONL (Chiarini et al., 2000). PACAP38 treatment was also effective against thapsigargin-induced damage, with 1 nM PACAP38 administered simultaneously with 10 nM thapsigargin effectively preventing damage to the photoreceptors of the ONL (Silveira et al., 2002).

#### Kainic acid

Kainic acid is a glutamate receptor agonist which is able to cause dose-dependent excitotoxicity-related injury to the retina. The intraocular injection of a low dose (6–20 nmol/retina) of this drug causes damage to the amacrine cells, with a 60 nmol/retina dose sufficient to cause degeneration of the bipolar and horizontal cells. Higher doses of kainic acid lead to the disappearance of the inner and outer plexiform layers in the chicken retina, while the photoreceptors and RGCs survived the treatment across the range of doses employed (Morgan and Ingham, 1981). The intravitreal administration of this drug in rats causes excitotoxic injury and cell death in the retina, especially in the INL, IPL, and CGL. PACAP (10 pmol) administered into the vitreous body 2 days before the kainic acid treatment resulted in a significantly lower incidence of cell death in the mentioned layers; however the co-administration of PACAP and kainic acid did not provide any protective effect in the retina (Seki et al., 2006b).

#### *N*-methyl-D-aspartate

*N*-methyl-D-aspartate (NMDA) is a selective glutamate receptor agonist, which is able to mimic the effect of glutamate. It is however specific to its NMDA receptor only, and it has no effect on other glutamate receptors such as the AMPA or kainite receptors. NMDA treatment induces an excitatory effect on several cell types in the CNS (Parsons et al., 1998; Watkins and Jane, 2006). In the retina, NMDA receptors are expressed by RGCs and amacrine cells (Fletcher et al., 2000), with the overstimulation of NMDA receptors causing retinal excitotoxicity which leads to neuronal cell death in the retina (Sucher et al., 1991), particularly in the GCL. NMDA is a drug commonly used to artificially create normotensive glaucoma models, and for testing anti-glaucoma drugs (Yamashita et al., 2011). PACAP could be a good candidate to combat NMDA-induced retinal injury because it is able to reduce the harmful effects associated with the intravitreal injection of NMDA. The effect of NMDA treatment was more severe in PACAP-null mice than in wild-type animals, with the decrease in the number of RGCs significantly greater on the first, third, and seventh days after the injection of 40 nmol NMDA in 2 µl saline (Endo et al., 2011). In this way, endogenous PACAP production can be seen to play an important role in the protection of retinal cells against NMDA-induced damage (Endo et al., 2011). PACAP38 co-injected with NMDA significantly increased the survival of RGCs both in wild-type and PACAP-null animals. The most effective concentration of PACAP38 in the case of treatment of the retina of wild-type mice was 100 pM (Endo et al., 2011).

#### Monosodium glutamate

Under normal conditions, the amino acid derivate glutamate is one of the most important excitatory neurotransmitters in the CNS. This molecule and its receptors are also present in the retina where they have an essential function. However, glutamate also plays a key role in neurological and retinal diseases, as well as in pathological conditions of the eye (Sucher et al., 1991; Danysz and Parsons, 2002). Monosodium glutamate (MSG) is one of the most commonly used drugs to induce retinal injury in different animal models or *in vitro* studies. In recent years, many papers have been published concerning MSG-induced retinal cytotoxicity, and treatments that are able to protect retinal cells against the harmful effects of increased glutamate levels. Several studies suggest that drugs such as glutamate receptor blockers, hormones, neuropeptides, or pre- and post-conditioning could be effective in treating pathological conditions where the glutamate level is increased (Lombardi and Moroni, 1994; Russo et al., 2008; Fernandez et al., 2009). PACAP could also be an interesting candidate for this given that it has been shown to be retinoprotective in several retinal degeneration models, as mentioned above, and is known to exert a protective effect against glutamate toxicity.

Newborn rat retinal cell primary cultures were exposed for 10 min to 1 mM glutamate which caused a significant decrease in their viability (Shoge et al., 1999). Treatment of the cells with either PACAP38 or PACAP27 was protective in a dose-dependent manner (1 nM to 1 µM) against this glutamate-induced damage, with a maximum protective effect observed at a concentration of 100 nM. After a 10-min treatment with PACAP, the level of PKA and the MAPK activity of the cells were elevated. PACAP6-38 and H-89 (a selective PKA inhibitor) were able to attenuate the positive effects of PACAP27 and PACAP38.

In addition to these in vitro results, some *in vivo* studies have suggested that PACAP is effective against glutamate toxicity. The intravitreal treatment with MSG of newborn rats causes severe degeneration in many layers of the retina. The average thickness of the retina was decreased, the IPL almost disappeared, and the INL and CGL layers seemed to fuse with each other (Tamas et al., 2004; Babai et al., 2005; Atlasz et al., 2009). Simultaneously administered PACAP38 and PACAP27 (100 pmol in 5 µl saline) were able to significantly attenuate the MSG-induced damage to the retina. Although the retina was thinner than that in untreated controls, damage to the affected layers of the retina was not so severe (Tamas et al., 2004; Babai et al., 2006; Atlasz et al., 2007). However, if more than one MSG injection was given into the vitreous body, one PACAP treatment was not enough to provide protection against the repetitive excitotoxic stimuli. The PACAP treatment was successful only in the case where PACAP (100 pmol) was administered at least two times in parallel with the MSG injection (Babai et al., 2005).

An important question concerns the combined and additive effects of different treatments against the excitotoxic effect of the glutamate. An interesting study published in 2010 addressed the effect of enriched environments to combat the effects of MSG. MSG was injected into the vitreous body of newborn rat pups and it was found that animals maintained in a bigger cage with colorful objects exhibited reduced glutamate damage compared with controls maintained under standard conditions. A similar or enhanced result was seen in cases where pups maintained under standard conditions were treated with PACAP (100 pmol diluted in 5 µl saline). However, the protective effect of the enriched environment and PACAP co-injection was not additive (Kiss et al., 2011).

Most studies published in the last few years examined the cytoprotective effect of PACAP from a morphological perspective, but no information is usually given about improvements in retinal function following the treatment of glutamate-induced damage. Until now, only one paper has been published in which the functional effects of MSG and PACAP treatments were measured by electroretinogram (ERG). Newborn rats were treated with MSG (2 mg/g bodyweight) administered subcutaneously on the first, fifth, and ninth days postnatal. PACAP (100 pmol diluted in 5 µl saline) was administered into the vitreous body in half of the pups on the same days. The ERG examination was performed 2 months later, with results showing that the subcutaneous MSG injection attenuated the ERG wave amplitude, and that PACAP treatment was able to significantly improve the functional performance of the damaged retina (Varga et al., 2011).

### DIRECT AND INDIRECT PATHWAYS

Pituitary adenylate cyclase-activating polypeptide suppresses neuronal activity in the CNS via direct and indirect pathways (Shioda et al., 2006). The direct pathway infers that PACAP affects target neurons expressing the PACAP receptor. As shown above, PAC1R was detected in GCLs and amacrine cells in the retina (Figure 1), suggesting that PACAP affects these kinds of neurons directly. On the other hand, PACAP is also expressed by RGCs and amacrine cells (Figure 1). It is known that endogenous PACAP has neuroprotective functions in the CNS (Ohtaki et al., 2006; Nakamachi et al., 2010; Reglodi et al., 2012). Indeed, PACAP knockout mouse deteriorate retinal damage in some retinopathy animal models (Endo et al., 2011; Szabadfi et al., 2012a), which implies that endogenous PACAP protects retinal neurons via auto- or paracrine mechanisms. Signaling pathways involving the protective function of PACAP on neurons have been elegantly summarized by Atlasz et al. (2010a).

In the case of indirect pathways, PACAP is known to stimulate the secretion of neuroprotective factors in the CNS (Somogyvari-Vigh and Reglodi, 2004), although little is known about PACAP’s indirect mode of action in the retina. IL-6 is recognized as a proinflammatory cytokine, but it acts as a neuroprotectant in the CNS (Loddick et al., 1998; Moidunny et al., 2010), and has been considered as a possible player in PACAP’s indirect neuroprotective pathway. PACAP administration significantly increases IL-6 mRNA and protein expression levels in the murine brain, with neurons and astrocytes identified as the source of PACAP-induced IL-6 secretion (Tatsuno et al., 1996; Ohtaki et al., 2006; Nakamachi et al., 2012). In the mouse brain, PACAP-induced neuroprotection is absent in the IL-6 knockout mouse, suggesting that PACAP suppresses neuronal damage via an IL-6-mediated pathway (Ohtaki et al., 2006). PACAP also has the potential to stimulate IL-6 secretion in the retina. The addition of PACAP to primary cultures of rat Müller cells significantly augmented IL-6 levels in the culture medium in a manner that was inhibited by PACAP6-38 treatment (Nakatani et al., 2006; Seki et al., 2006a). Indeed, PAC1R protein was detected in rat primary cultures of Müller cells (Kubrusly et al., 2005). These data suggest that IL-6 released from Müller cells may mediate PACAP-induced retinal protection. Recent reports suggest that Müller cells secrete many types of neurotrophic factors, growth factors, and cytokines, such as BDNF and neurotrophin-3, glial cell-line derived neurotrophic factor (GDNF), neurturin, ciliary neurotrophic factor (CNTF), endothelin-2, leukemia inhibitory factor (LIF), basic fibroblast growth factor (bFGF), and prostaglandin E2 (Bringmann et al., 2009). These factors may be related to the neuroprotective effect of PACAP as well as IL-6. Furthermore, microglial cells exist in the retina, and infiltrating macrophages were identified in the retina after injury (Chen et al., 2002; Davies et al., 2006). This microglia/macrophage system has been considered as a regulator of immunity and inflammation after retinal damage (Wraith and Nicholson, 2012). Further study focusing on retinal glial cells in the mechanism of PACAP neuroprotection will provide new insights of the protective network involving PACAP.

## CONCLUSION AND FUTURE PERSPECTIVES

In the clinical setting, retinal neuropathies are a major cause of visual impairment that are yet to have a definitive cure. Alternative therapeutic strategies for addressing optic neuropathies are therefore required. Neurotrophic factors such as BDNF, ciliary neurotrophic factor, and glial cell line-derived neurotrophic factor have been shown to ameliorate RGC damage in an animal model of retinopathy (Johnson et al., 2011). However, the actions of these growth factors have not yet been well elucidated to enable them to be considered as target candidates of neuroprotective drugs to treat retinal neuropathies. In this review, we have shown that PACAP consistently exerts a potent protective effect against neural damage in a broad range of retinal diseases. As future studies involving PACAP will likely shift to obtaining a fuller understanding of the mechanisms underlying such protective functions, an important aspect of this will be to examine pathways involving glial cells. These insights will help in the development of new neuroprotective strategies to treat retinal neuropathies.

## Conflict of Interest Statement

The authors declare that the research was conducted in the absence of any commercial or financial relationships that could be construed as a potential conflict of interest.
